# Cyclooxygenase‐1 and ‐2 modulate sweating but not cutaneous vasodilation during exercise in the heat in young men

**DOI:** 10.14814/phy2.13844

**Published:** 2018-09-02

**Authors:** Naoto Fujii, Olivia L. Pastore, Gregory W. McGarr, Robert D. Meade, Brendan D. McNeely, Takeshi Nishiyasu, Glen P. Kenny

**Affiliations:** ^1^ Human and Environmental Physiology Research Unit University of Ottawa Ottawa Canada; ^2^ Faculty of Health and Sport Sciences University of Tsukuba Tsukuba Japan

**Keywords:** cAMP, endothelium‐dependent vasodilation, microcirculation, NSAIDs, prostanoids, thermoregulation

## Abstract

We recently reported that the nonselective cyclooxygenase (COX) inhibitor ketorolac attenuated sweating but not cutaneous vasodilation during moderate‐intensity exercise in the heat. However, the specific contributions of COX‐1 and COX‐2 to the sweating response remained to be determined. We tested the hypothesis that COX‐1 but not COX‐2 contributes to sweating with no role for either COX isoform in cutaneous vasodilation during moderate‐intensity exercise in the heat. In thirteen young males (22 ± 2 years), sweat rate and cutaneous vascular conductance were measured at three forearm skin sites that were continuously treated with (1) lactated Ringer's solution (Control), (2) 150 *μ*mmol·L^−1^ celecoxib, a selective COX‐2 inhibitor, or (3) 10 mmol L^−1^ ketorolac, a nonselective COX inhibitor. Participants first rested in a non heat stress condition (≥85 min, 25°C) followed by a further 70‐min rest period in the heat (35°C). They then performed 50 min of moderate‐intensity cycling (~55% peak oxygen uptake) followed by a 30‐min recovery period. At the end of exercise, sweat rate was lower at the 150 *μ*mol·L^−1^ celecoxib (1.51 ± 0.25 mg·min^−1^·cm^−2^) and 10 mmol·L^−1^ ketorolac (1.30 ± 0.30 mg·min^−1^·cm^−2^) treated skin sites relative to the Control site (1.89 ± 0.27 mg·min^−1^·cm^−2^) (both *P* ≤ 0.05). Additionally, sweat rate at the ketorolac site was attenuated relative to the celecoxib site (*P* ≤ 0.05). Neither celecoxib nor ketorolac influenced cutaneous vascular conductance throughout the experiment (both *P* > 0.05). We showed that both COX‐1 and COX‐2 contribute to sweating but not cutaneous vasodilation during moderate‐intensity exercise in the heat in young men.

## Introduction

Nonsteroidal antiinflammatory drugs (NSAIDs) are widely utilized by the general population for management of pain, fever, and inflammation. NSAIDs function primarily through inhibition of the cyclooxygenase (COX) enzyme. While the prevalence of NSAID use is relatively high in older adults, they are also commonly used by young individuals to manage pain and/or inflammation, especially in those engaged in competitive athletics (Warner et al. 2002; Da Silva et al. 2011). Exercise results in hyperthermia, which is exacerbated under high ambient temperature conditions. Previous work has evaluated how oral intake of NSAIDs modulates body temperatures and/or the heat loss responses of sweating and cutaneous vasodilation (Charkoudian and Johnson 1999; Bradford et al. 2007; Bruning et al. 2013). However, NSAID ingestion acts systemically, inducing both local and central effects. To better understand the mechanisms by which NSAIDs modulate sweating and cutaneous vasodilation during exercise in the heat, it is important to isolate the influence of local COX inhibition on these heat loss responses, independent of any systemic effects.

The local mechanisms underpinning the heat loss responses have been extensively studied and several key modulators have been identified including nitric oxide synthase (Kellogg et al. 1995; McNamara et al. 2014; Meade et al. 2015; Fujii et al. 2016), K^+^ channels (Brunt et al. 2013; Louie et al. 2016, 2017), neurotransmitters released from sympathetic cholinergic nerves (Kellogg et al. 1998, 2012), and COX (McCord et al. 2006; Fujii et al. 2014b). Regarding the latter, we recently reported that nonselective COX inhibition (via ketorolac) attenuated sweat rate in young habitually active adults during moderate‐intensity exercise in the heat (Fujii et al. 2014b). However, the specific role of each COX isoform (COX‐1 and COX‐2) remains unclear. Human eccrine sweat glands possess COX‐1 with no clear expression of COX‐2 (Muller‐Decker et al. 1999), suggesting that COX‐1 alone may contribute to the sweating response. However, this possibility has not yet been evaluated. Elucidating which COX isoform contributes to the sweating response during exercise in the heat is important, since although most NSAIDs block both COX isoforms, some block either one (e.g., a low dose of aspirin more selectively inhibits COX‐1) (Ornelas et al. 2017).

In contrast to the sweating response, we recently demonstrated that dual blockade of COX‐1 and COX‐2 had no effect on cutaneous vasodilation during moderate‐intensity exercise in the heat (Fujii et al. 2014b). While this finding may be interpreted as a lack of a role for either COX isoform in modulating cutaneous vasodilation under these conditions, it is also possible that COX‐1 and COX‐2 confer opposing vasoactive effects (i.e., vasoconstriction vs. vasodilation), thus warranting further scrutiny. Indeed, it is believed that COX‐1 predominantly produces the vasoconstrictor thromboxane A2, whereas COX‐2 creates the vasodilator prostacyclin (Bates and Lau 2005; Rovati et al. 2010; Feletou et al. 2011). Assessing the effects of selective COX inhibitors on vascular responses during heat stress would act to clarify the role of this enzyme in the cutaneous vasodilatory response to exercise‐induced heat stress.

The purpose of this study was to evaluate the hypothesis that in young men, COX‐1 but not COX‐2 contributes to the sweating response during moderate‐intensity exercise in the heat. We also hypothesized that neither COX‐1 nor COX‐2 would influence cutaneous vasodilation.

## Materials and Methods

### Ethical approval

The current study was approved by the University of Ottawa Health Sciences and Science Research Ethics Board and complied with the guidelines set forth by the Declaration of Helsinki. All participants gave verbal and written informed consent prior to participation.

### Participants

Thirteen young habitually active males volunteered to participate in the study. We tested males only to avoid any potential sex‐related differences in the regulation of cutaneous vasodilation and sweating (Gagnon et al. 2013; Greaney et al. 2014; Fujii et al. 2015a). All participants were screened for cardiovascular, respiratory, metabolic, and skin diseases before participating. Participants were nonsmoking, and not taking any prescription medications. Mean (±SD) age, body mass, height, surface area, and peak oxygen uptake were: 22 ± 2 years, 80.0 ± 8.8 kg, 1.79 ± 0.07 m, 1.99 ± 0.13 m^2^, and 47.9 ± 5.1 mL·kg^−1^·min^−1^, respectively.

### Experimental design

All participants completed one screening and one experimental session. Prior to each session, participants were instructed to refrain from engaging in heavy exercise and consuming over‐the‐counter medications including NSAIDs (e.g., aspirin and ibuprofen have half‐lives of less than 6 h (Schiodt et al. 2002)), vitamins, and minerals for a minimum of 48 h, caffeinated beverages or alcohol for at least 12 h, and food for 2 h.

During the screening session, body mass was measured using a digital weight scale platform (model CBU1500X; Mettler Toledo, Schwerzenbach, Switzerland) with a weighing terminal (model IND560; Mettler Toledo Inc., Mississauga, ON, Canada), while height was assessed using an eye‐level physician stadiometer (Detecto, model 2391, Webb City, MO). These two measurements were used to calculate body surface area using the equation developed by Du Bois and Du Bois (Du Bois and Du Bois 1916). Body fat percentage was estimated through body density measured via the hydrostatic weighing technique. Peak oxygen uptake was determined through an incremental cycling exercise protocol performed on a semirecumbent cycling ergometer (Corival Recumbent, Lode B.V., Groningen, the Netherlands). Participants maintained a pedaling rate of 60–100 rpm at a starting resistance of 80 W, which was subsequently increased by 20 W·min^−1^ until volitional fatigue. Ventilatory and metabolic data were collected using an automated indirect calorimetry system (Medgraphic Ultima, Medical Graphic Corporation, St Paul, MN).

The experimental session was performed on a day following and separated from the screening session by a minimum of 48 h. Upon arrival at the laboratory, participants voided their bladder, after which pretrial body mass was measured on a weighing terminal (Mettler Toledo Inc.). Participants were then seated on a surgical bed in a non heat stress ambient temperature condition of 25°C. Three microdialysis fibers (30 kDa cutoff, 10 mm membrane; MD2000, Bioanalytical Systems, West Lafayette, IN) were inserted into the dermal layer of the skin on the left dorsal forearm. Under aseptic conditions, a 25‐gauge needle was inserted into the skin (~2.5 cm in length). The microdialysis fiber was then threaded through the lumen of the needle, after which the needle was removed leaving the fiber embedded in the forearm skin. Each fiber was secured with surgical tape, and separated from adjacent fibers by 2–4 cm.

After the insertion of the fibers, the participant was transferred to a thermal chamber (Can‐Trol Environmental Systems, Markham, ON, Canada) regulated to 25°C and 20% relative humidity where they rested while seated on a semirecumbent cycle ergometer (Corival Recumbent, Lode B.V.). At this time, the perfusion of the pharmacological agents with a micro‐infusion pump (Model 400, CMA Microdialysis, Solna, Sweden) was initiated at each of the three microdialysis sites at a rate of 4 *μ*L·min^−1^. The sites were perfused with either (1) lactated Ringer's solution (Baxter, Deerfield, IL) (Control), (2) 150 *μ*mol·L^−1^ celecoxib (Cayman Chemical, Ann Arbor, MI), a selective COX‐2 inhibitor, or (3) 10 mmol·L^−1^ ketorolac (Sigma‐Aldrich, St. Louis, MO), a nonselective COX inhibitor. Given that celecoxib is water‐insoluble, the organic solvent dimethyl sulfoxide (Sigma‐Aldrich) was required to make celecoxib solution. In our pilot work, we found 5% dimethyl sulfoxide to have no effect on sweating or cutaneous vascular responses relative to a Control site (i.e., only lactated Ringer's solution) during exercise in the heat. Therefore, any difference between the control (lactated Ringer's solution) and 150 *μ*mol·L^−1^ celecoxib sites should reflect a COX‐2 inhibition effect in the present study. Previous work has demonstrated that ~150 *μ*mol·L^−1^ celecoxib maximally inhibits COX‐2 (Tacconelli et al. 2002). The concentration of ketorolac was based on previous microdialysis work (Holowatz et al. 2005, 2009; Kellogg et al. 2005; McCord et al. 2006; Medow et al. 2007; Yamazaki et al. 2007; Fujii et al. 2013, 2014b; Kutz et al. 2015). Drug perfusion continued for a minimum of 75 min in order to ensure maximal inhibition of COX. This time period is sufficient to ensure that any redness associated with fiber insertion had subsided (Anderson et al. 1994). Drug perfusion continued throughout the entire experimental protocol to ensure the sustained blockade of COX.

Following the ≥75 min habituation period in a non heat stress ambient temperature condition of 25°C, participants were monitored for an additional 10 min to acquire baseline measurements. Thereafter, room temperature was increased to 35°C within 10 min. Thereafter the participants remained resting on the semirecumbent cycle ergometer for at least 70 min. Resting measurements at 35°C were recorded during the final 10 min. Participants then performed 50‐min cycling at a moderate intensity (equivalent to ~55% of their predetermined peak oxygen uptake), which was followed by a 30‐min recovery period. This exercise intensity was chosen to induce a significant increase in body core temperature and therefore increases in sweating and cutaneous perfusion, but it was low enough to ensure that a COX‐dependent sweating would occur (Fujii et al. 2014b). Thereafter, 50 mmol·L^−1^ sodium nitroprusside (Sigma‐Aldrich) was infused for 20–25 min at all sites at a rate of 6 *μ*L·min^−1^ until maximum values for cutaneous perfusion were achieved for a minimum of 2 min. Upon completion of the experimental session, the participant's body mass was measured.

### Measurements

A sweat capsule specifically designed for use with an intradermal microdialysis probe that covers an area of 1.1 cm^2^ (Meade et al. 2016) was placed directly over the center of each microdialysis membrane. The sweat capsules were attached to the skin with adhesive rings and topical skin glue (Collodion HV, Mavidon Medical products, Lake Worth, FL). Dry compressed air from gas tanks located in the temperature‐controlled chamber was supplied to each capsule at a constant flow rate, while water content of the effluent air was measured with a capacitance hygrometer (model HMT333, Vaisala, Helsinki, Finland). Long vinyl tubes were used for connections between the gas tank and the sweat capsule (inlet), and between the sweat capsule and the capacitance hygrometer (outlet). Local forearm sweat rate was measured continuously (5 sec sampling rate) and calculated from the water content of the effluent air multiplied by the flow rate and normalized for the skin surface area under the capsule (mg·min^−1^·cm^−2^).

Cutaneous blood flow (expressed in perfusion units) was measured at three local sites on the forearm using laser‐Doppler flowmetry (PeriFlux System 5000, Perimed, Stockholm, Sweden) at a sampling rate of 32 Hz. Integrated seven‐laser array laser‐Doppler probes (Model 413, Perimed) were situated directly above the centre of the membrane of the microdialysis fibers. Cutaneous vascular conductance (CVC) was evaluated as laser‐Doppler flux, an index of cutaneous blood flow, divided by mean arterial pressure, and expressed as a %max of values from the maximal absolute CVC protocol. Blood pressure was determined every 5 min using a manual mercury column sphygmomanometer (Baumonometer Standby Model, WA Baum Co, Copiague, NY). Mean arterial pressure was calculated as diastolic arterial pressure plus one‐third the difference between systolic and diastolic pressures (i.e., pulse pressure).

Body core temperature was measured by aural canal temperature (Braun Thermoscan Pro 6000, Welch Allyn, Skaneateles Falls, NY), which has been shown to closely track changes in esophageal temperature, especially in the heat (Taylor et al. 2014). Skin temperature was measured continuously at four sites (calf, quadriceps, chest, and biceps) using thermocouple discs (Concept Engineering, Old Saybrook, CT) attached to the skin with adhesive rings and surgical tape. Mean skin temperature was estimated as a weighted mean using the local skin temperatures of the calf (20%), quadriceps (20%), biceps (30%), and chest (30%) (Hardy and Dubois 1938). Skin temperature data was collected using a data acquisition module (Model 34970A; Agilent Technologies Canada Inc., Mississauga, ON, Canada). The data was displayed and recorded using LabVIEW software (National Instruments, Austin, TX). Heart rate was measured continuously using a Polar coded WearLink and transmitter, Polar RS400 interface, and Polar Trainer 5 software (Polar Electro, Kempele, Finland).

Metabolic energy expenditure was measured via indirect calorimetry using electrochemical gas analyzers (AMETEK model S‐3A/1 and CD3A, Applied Electrochemistry, Pittsburgh, PA) to determine O_2_ and CO_2_ concentrations of expired air. Participants wore a fitted facemask (Model 7600 V2, Hans‐Rudolph, Kansas City, MO) attached to a 2‐way T‐shape nonrebreathing valve (Model 2700, Hans‐Rudolph). Oxygen uptake and respiratory exchange ratio were calculated from O_2_ and CO_2_ concentrations in expired air, and sampled every 30 sec to estimate metabolic rate.

The urine sample obtained at the start of the experimental protocol was analyzed with a total solids refractometer (Model TS400, Reichter Inc., Depew, NY) to evaluate urine specific gravity, an index of body fluid status.

### Data analysis

Based on sweat rate (Stapleton et al. 2014) and cutaneous vascular (Fujii et al. 2014a) responses obtained in our previous work, with 80% power and a significance level of 0.05, a minimal sample size of *n* = 8 for sweat rate and *n* = 9 for CVC was determined. For all variables, a 5‐min average was taken for resting periods [under non heat stress (25°C) and heat stress (35°C) conditions] and at 10‐min intervals during exercise and the subsequent recovery. For the measurement of maximal cutaneous blood flow during the sodium nitroprusside treatment, responses were averaged over the 2‐min plateau interval. Blood pressure and aural canal temperature were manually recorded every 5 min, and an average of the two values measured during a 10‐min interval were used for data analysis. Further, due to technical issues some measurements were not recorded for some participants (numbers of participants excluded for data analysis are indicated in parentheses): sweat rate (*n* = 1), body core (*n* = 1) and mean skin (*n* = 3) temperatures, and heart rate (*n* = 1).

### Statistical analyses

Sweat rate and CVC data were analyzed with a two‐way repeated‐measures analysis of variance (ANOVA) with the factors of treatment site (Control, 150 *μ*mol·L^−1^ celecoxib, 10 mmol·L^−1^ ketorolac) and time point (resting at 25°C and 35°C and every 10 min during and following exercise). Maximum absolute CVC (perfusion units·mmHg^−1^) was analyzed with a one‐way repeated‐measures ANOVA with a factor of treatment site. Secondary variables (body core and mean skin temperatures, heart rate, and mean arterial pressure) were analyzed with a one‐way repeated‐measures ANOVA with a factor of time point (resting at 25°C and 35°C as well as end‐exercise and end‐recovery). When a significant interaction or main effect was detected, post hoc multiple comparisons were carried out using a Holm‐Bonferroni correction. In addition, Student's *t*‐tests were employed where applicable. The level of significance for all analyses was set at *P* ≤ 0.05. All values are reported with a mean ± 95% confidence interval (1.96 ×  standard error of the mean). Statistical analyses were conducted using SPSS 25 (IBM, Armonk, NY).

## Results

### Sweat rate

**Figure 1:**
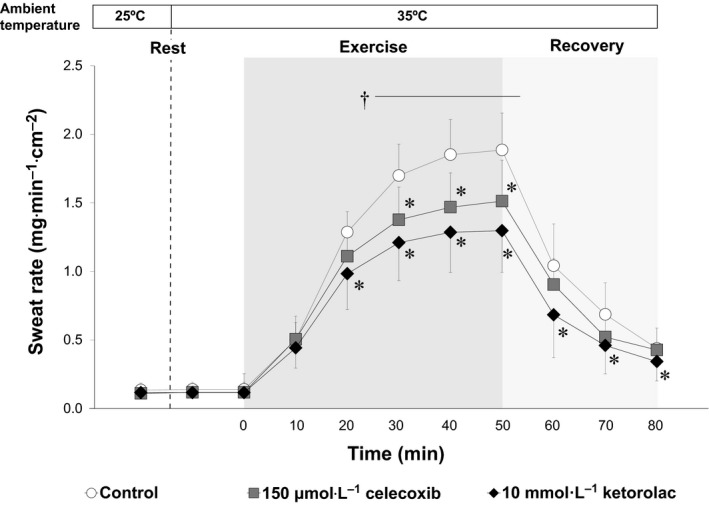
Sweat rate recorded at rest during a non heat stress (25°C) and heat stress (35°C) condition as well as during (dark grey shaded area) and following (light grey shaded area) exercise in the heat (35°C). Three intradermal forearm skin sites were continuously treated with either: (1) lactated Ringer's solution (Control), (2) 150 *μ*mol·L^−1^ celecoxib (specific COX‐2 inhibitor), or (3) 10 mmol·L^−1^ ketorolac (COX‐1 and COX‐2 inhibitor). * Control versus drug treated site (*P* ≤ 0.05); † 150 *μ*mol·L^−1^ celecoxib versus 10 mmol·L^−1^ ketorolac (*P* ≤ 0.05). All values are expressed as mean ± 95% confidence interval (*n* = 12). Resting values are presented as the average of the final 5‐min resting values of the 10‐min and 70‐min resting period at 25°C and 35°C respectively. All values during exercise and recovery periods were obtained by averaging over the final 5‐min of each time interval.

Sweat rate during rest in both non heat stress (25°C) and heat stress (35°C) conditions did not differ between skin sites (*P* > 0.05, Fig. 1). During exercise, sweat rates at all skin sites were increased relative to preexercise rest in the heat. Compared to Control, both the administration of celecoxib and ketorolac attenuated sweat rate during exercise in the heat (*P* ≤ 0.05, Fig. 1). Further, sweat rate at the ketorolac treated site was lower than that at the celecoxib site (*P* ≤ 0.05, Fig. 1). During postexercise recovery in the heat, sweat rate remained reduced relative to Control by ketorolac only (*P* ≤ 0.05, Fig. 1).

### Cutaneous vascular response

**Figure 2:**
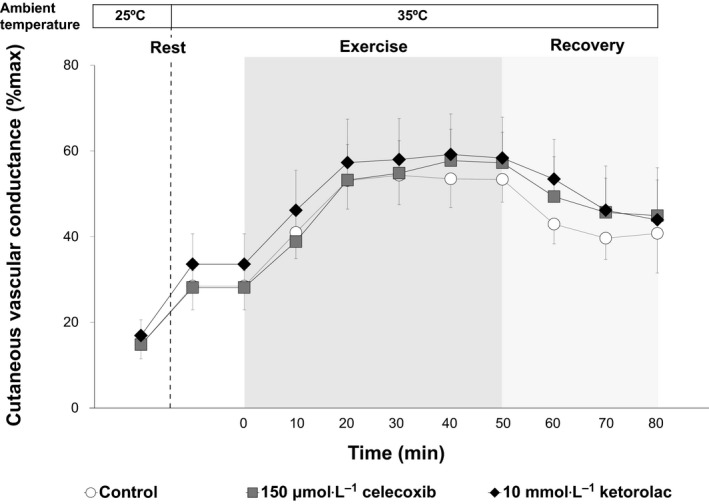
Cutaneous vascular conductance recorded at rest during a non heat stress (25°C) and heat stress (35°C) condition as well as during (dark grey shaded area) and following (light grey shaded area) exercise in the heat (35°C). Three intradermal forearm skin sites were continuously treated with either: (1) lactated Ringer's solution (Control), (2) 150 *μ*mol·L^−1^ celecoxib (specific COX‐2 inhibitor), or (3) 10 mmol·L^−1^ ketorolac (COX‐1 and COX‐2 inhibitor). Cutaneous vascular conductance did not differ between sites (*P* > 0.05). All values are expressed as mean ± 95% confidence interval (*n* = 13). Resting values are presented as the average of the final 5‐min resting values of the 10‐min and 70‐min resting period at 25°C and 35°C respectively. All values during exercise and recovery periods were obtained by averaging over the final 5‐min of each time interval.

Cutaneous vascular conductance did not differ between skin sites throughout the experimental protocol (Fig. 2). Likewise, between‐site differences in maximal absolute CVC were not observed (Table 1).

**Table 1:** Absolute maximal cutaneous vascular conductance at the three skin sites

	(perfusion units**·**mmHg^−1^)
Control	1.94 ± 0.40
150 *μ*mol·L^−1^ celecoxib	2.19 ± 0.36
10 mmol·L^−1^ ketorolac	1.93 ± 0.32

Values are expressed as mean ± 95% confidence interval (*n* = 13). Celecoxib is a specific COX‐2 inhibitor. Ketorolac is a nonselective COX inhibitor. There were no between‐site differences (*P* = 0.89 for a main effect of treatment site).

### Body temperatures and cardiovascular responses

In comparison to the non heat stress resting condition, body core (36.67 ± 0.12°C) and mean skin (31.86 ± 0.23°C) temperatures increased by 0.27 ± 0.06 and 2.30 ± 0.23°C, respectively during rest in the heat (35°C) (both *P* ≤ 0.05). This was paralleled by increases in heart rate and mean arterial pressure from 67 ± 5 to 72 ± 6 beats·min^−1^ and from 86 ± 4 to 88 ± 5 mmHg, respectively (both *P* ≤ 0.05). At end‐exercise, body core and mean skin temperatures increased by 0.76 ± 0.20 and 1.24 ± 0.17°C above preexercise resting levels in the heat with concomitant increases in heart rate (154 ± 10 beats·min^−1^) and mean arterial pressure (102 ± 7 mmHg) (all *P* ≤ 0.05). During the postexercise recovery period, body core temperature (37.28 ± 0.15°C) and heart rate (95 ± 5 beats·min^−1^) remained elevated above resting values in the heat (both *P* ≤ 0.05); however, mean skin temperature (34.49 ± 0.26°C) had returned to preexercise resting level in the heat (*P* > 0.05), and mean arterial pressure (83 ± 5 mmHg) was reduced (*P* ≤ 0.05).

### Body weight and urine specific gravity

Following the experiment, body weight was reduced by 1.3 ± 0.4% from the pre experiment value (*P* ≤ 0.05). Urine specific gravity evaluated before starting the experiment was 1.017 ± 0.005. Consequently, participants were adequately hydrated (urine specific gravity <1.025; (Kenefick and Cheuvront 2012)) prior to the experiment.

## Discussion

We evaluated the relative contributions of COX‐1 and COX‐2 to the regulation of sweating and cutaneous vasodilation in young adults during exercise in the heat. We showed that the lone inhibition of COX‐2, as well as the combined inhibition of COX‐1 and COX‐2, reduced sweat rate during moderate‐intensity exercise in the heat. The reduction in sweat rate achieved by the combined inhibition of both isoforms was greater compared to that achieved by the selective COX‐2 inhibition. In contrast to our first hypothesis, these results suggest that both COX‐1 and COX‐2 contribute to the sweating response in young habitually active men during moderate‐intensity exercise in the heat. Consistent with our second hypothesis, we observed no role for either COX isoform in the regulation of cutaneous perfusion.

### Sweating

We show that COX‐2 contributes to the regulation of sweating during moderate‐intensity exercise in the heat (Fig. 1). This is in contrast to a previous report that was unable to confirm the presence of COX‐2 in human eccrine sweat glands in vitro (Muller‐Decker et al. 1999). However, that study did not provide the patient characteristics from which the skin biopsies were obtained (Muller‐Decker et al. 1999). We recently showed that aging attenuates COX‐dependent sweating during exercise (Fujii et al. 2015b). Thus, the lack of histological evidence for COX‐2 in human eccrine sweat glands (Muller‐Decker et al. 1999) may have been due to the age of the subjects from which the skin biopsies were obtained. It is also important to note that the above human eccrine sweat gland study reported the existence of COX‐2 in keratinocytes (Muller‐Decker et al. 1999). It may be that prostaglandins produced in keratinocytes via COX‐2 are able to migrate to eccrine sweat glands via the interstitial space and ultimately influence the sweating response during exercise in the heat. Alternatively, a previous in vitro study reported that COX‐2 (generally known as inducible COX) can be upregulated by heat stress (Rossi et al. 2012). This may indicate that an increase in mean skin temperature associated with endogenous heat production during exercise may upregulate COX‐2 to influence the sweating response under these conditions. Consistent with this possibility, we showed that the inhibition of COX‐2 only attenuated the sweating response during the later stages of exercise (≥30 min) when mean skin temperature had reached sustained elevated levels above baseline resting values.

In agreement with our previous work (Fujii et al. 2014b), we demonstrated that nonselective inhibition of both COX‐1 and COX‐2 (via ketorolac) reduced sweat rate during moderate‐intensity (~55% peak oxygen uptake) exercise in the heat (Fig. 1). To evaluate the separate influence of COX‐1 on the sweating response, we compared sweat rates between the combined inhibition of COX‐1 and COX‐2 (ketorolac) and COX‐2 inhibition only (celecoxib). During exercise we observed a lower sweat rate at the nonselective inhibition site compared to that at the COX‐2 inhibited site (Fig. 1). While the overall contribution of COX‐2 appears to be greater, this finding suggests that COX‐1 is also involved in regulating sweating during moderate‐intensity exercise in the heat. In this context, our findings support previous work, which identified the presence of COX‐1 in human eccrine sweat glands (Muller‐Decker et al. 1999). Based on the end exercise data in Fig. 1, combined COX‐1 and COX‐2 explains ~30% of the sweating response during exercise in the heat, and this relative contribution is similar to that of nitric oxide synthase and the Na‐K‐2Cl co‐transporter (Fujii et al. 2014b; Louie et al. 2016). Our previous work demonstrated that COX and nitric oxide synthase work in an interactive manner to promote the sweating response during exercise in the heat (Fujii et al. 2014b). Whether the Na‐K‐2Cl co‐transporter modulates the sweating response interactively with COX and nitric oxide synthase requires future study.

### Cutaneous vascular response

In contrast to the sweating response, the present study demonstrated that neither COX isoform plays a role in mediating cutaneous vasodilation during exercise in the heat, as well as during pre and postexercise resting conditions in the heat (Fig. 2). We are not aware of any evidence directly or indirectly explaining this differential effect of COX inhibition on sweating and cutaneous perfusion. Several factors have been shown to influence COX‐dependent mechanisms including prostaglandin receptors, adenylyl cyclase (an enzyme that forms the important second messenger cAMP in the COX pathways), and phosphodiesterase (an enzyme that degrades cAMP) (Klein et al. 2007). Consequently, future studies are therefore necessary to elucidate the separate and combined influences of these modulators in the regulation of end‐organ function of the cutaneous vasculature and eccrine sweat gland, and therefore skin blood flow and sweating during rest and exercise in the heat.

Although we detected no role of COX in cutaneous vasodilation during rest, moderate‐intensity exercise, or postexercise recovery in the heat (35°C) in the present study (Fig. 2) and in our previous work (Fujii et al. 2014b), McCord et al. (2006) did demonstrate a role of COX in cutaneous vasodilation during a passive heat stress induced by whole‐body heating with a water‐perfused suit. The reasons underlying the disparate results between our collective findings and those by McCord et al. (2006) remain unclear. However, differences in skin temperature may in part underlie these different findings. For example, in the present study, mean skin temperature remained near levels equal to ambient temperature (~35°C). In contrast, McCord et al. (2006) employed a passive heating protocol wherein water temperature of the tube‐lined perfusion suit was markedly increased to heat the body. Such a maneuver typically causes mean skin temperature to reach ~39°C (Kellogg et al. 2008, 2009; Brunt et al. 2013). Since the level of heat stress can influence regulation of COX‐2 (Rossi et al. 2012), the greater mean skin temperature in the study by McCord et al. (2006) may have led to a greater COX‐2 activation, thereby resulting in a more robust contribution of this enzyme to the regulation of cutaneous vasodilation. Further studies are required to better understand the influence of skin temperature on COX‐dependent cutaneous vasodilation.

We showed that dual blockade of COX‐1 and COX‐2 with ketorolac did not affect cutaneous vasodilation during exercise in the heat. This response may indicate that COX‐1 and COX‐2 have counteracting vasoactive influences, causing no effect on cutaneous vascular tone during exercise in the heat. In line with this notion, it is thought that COX‐1 chiefly produces the vasoconstrictor thromboxane A2, while COX‐2 creates the vasodilator prostacyclin (Bates and Lau 2005; Rovati et al. 2010; Feletou et al. 2011). However, in the current study we excluded this possibility, as inhibition of COX‐2 alone had no effect on cutaneous vasodilation during exercise in the heat (Fig. 2). Taken together, our findings demonstrate that neither COX‐1 nor COX‐2 appear to provide any vasoactive effects in the skin during exercise in the heat, thereby confirming that these isoforms are not actively producing counteracting vasoactive substances under the study conditions tested. Further work is required to assess this response at increasing levels of heat stress (as defined by increasing environmental and/or exercise‐induced heat loads).

### Perspective and significance

Sweating and cutaneous vasodilation are crucial for heat dissipation and the regulation of body core temperature during exercise, especially in the heat. An impairment in the body's capacity to dissipate heat can result in a potentially dangerous increase in body core temperature. In this context, the use of NSAIDs may compromise heat dissipation during exercise in the heat. NSAIDs are COX inhibitors that are widely used for reducing pain, fever, and inflammation. Although most of these drugs block both COX isoforms, some selectively block either COX‐1 or COX‐2 (e.g., low dose aspirin more selectively inhibits COX‐1). Given the widespread use of NSAIDs by young individuals engaging in regular exercise (Warner et al. 2002; Da Silva et al. 2011), advancing our understanding of the role of COX‐1 and/or COX‐2 inhibition on the regulation of heat loss responses in this population is important. Our findings suggest that NSAIDs may attenuate the sweating response to an exercise‐induced heat stress. Consequently, young adults may be at greater risk of developing a heat‐related injury. This risk may be exacerbated with the consumption of drugs that block both isoforms (e.g., ibuprofen), unlike NSAIDs which selectively block either the COX‐1 or ‐2 isoform (e.g., celecoxib, low‐dose aspirin). Given we locally inhibited these COX isoforms, our results may be more applicable to conditions in which topical NSAIDs are used for the treatment of muscle pains (Manoukian et al. 2017; Yancey and Gill 2017). Further studies are required to determine if the use of oral and topical NSAIDs cause similar attenuations in heat loss responses. Additionally, work must be conducted to determine if these responses remain intact in older adults. As noted above, NSAIDs are commonly prescribed to older adults for the treatment of pain and inflammation as well as for the management of cardiovascular disease. In view of the fact that a previous work demonstrated significant impairments in the capacity of older adults (≥40 years) to dissipate heat (Larose et al. 2013), studies must be conducted to determine if the use of NSAIDs may aggravate this response.

## Conclusion

We show that in young habitually active men, both COX‐1 and COX‐2 contribute to sweating, but not cutaneous vasodilation, during moderate‐intensity exercise in the heat.

## Conflict of Interests

None declared.
